# Headcase is a Repressor of Lamellocyte Fate in *Drosophila melanogaster*

**DOI:** 10.3390/genes10030173

**Published:** 2019-03-05

**Authors:** Gergely I. B. Varga, Gábor Csordás, Gyöngyi Cinege, Ferenc Jankovics, Rita Sinka, Éva Kurucz, István Andó, Viktor Honti

**Affiliations:** 1 Laboratory of Immunology, Institute of Genetics, Biological Research Centre of the Hungarian Academy of Sciences, 6726 Szeged, Hungary; varga.gergely@brc.mta.hu (G.I.B.V.); cgabor@uni-koeln.de (G.C.); cinege.gyongyi@brc.mta.hu (G.C.); kurucz.eva@brc.mta.hu (É.K.); 2 Laboratory of *Drosophila* Germ Cell Differentiation, Institute of Genetics, Biological Research Centre of the Hungarian Academy of Sciences, 6726 Szeged, Hungary; jankovics.ferenc@brc.mta.hu; 3 Department of Genetics, Faculty of Science and Informatics, University of Szeged, 6726 Szeged, Hungary; rsinka@bio.u-szeged.hu

**Keywords:** *Drosophila*, hemocyte, blood cell, innate immunity, differentiation, hematopoiesis, niche

## Abstract

Due to the evolutionary conservation of the regulation of hematopoiesis, *Drosophila* provides an excellent model organism to study blood cell differentiation and hematopoietic stem cell (HSC) maintenance. The larvae of *Drosophila melanogaster* respond to immune induction with the production of special effector blood cells, the lamellocytes, which encapsulate and subsequently kill the invader. Lamellocytes differentiate as a result of a concerted action of all three hematopoietic compartments of the larva: the lymph gland, the circulating hemocytes, and the sessile tissue. Within the lymph gland, the communication of the functional zones, the maintenance of HSC fate, and the differentiation of effector blood cells are regulated by a complex network of signaling pathways. Applying gene conversion, mutational analysis, and a candidate based genetic interaction screen, we investigated the role of Headcase (Hdc), the homolog of the tumor suppressor HECA in the hematopoiesis of *Drosophila*. We found that naive loss-of-function *hdc* mutant larvae produce lamellocytes, showing that Hdc has a repressive role in effector blood cell differentiation. We demonstrate that *hdc* genetically interacts with the Hedgehog and the Decapentaplegic pathways in the hematopoietic niche of the lymph gland. By adding further details to the model of blood cell fate regulation in the lymph gland of the larva, our findings contribute to the better understanding of HSC maintenance.

## 1. Introduction

Hematopoiesis is a sequence of strictly regulated events, during which mature blood cells differentiate from hematopoietic stem cells (HSCs) [1,2]. The ability of HSCs to self-renew is maintained by a complex network of signaling pathways in a specific microenvironment, the hematopoietic niche [3,4,5]. As improper regulation of HSC function leads to development of leukemias [6,7], the maintenance of the homeostatic balance between HSC self-renewal and blood cell differentiation is essential for the survival of the organism.

Due to the similarities of its immune system to that of vertebrates, *Drosophila* is extensively used as a model system, via which to study hematopoiesis. Similar to their vertebrate counterparts, the blood cells of *Drosophila*, the hemocytes, differentiate in several waves throughout ontogenesis and are localized in dedicated hematopoietic compartments in each developmental stage. Such hemocyte compartments in the larva are the lymph gland, the sessile tissue, and the circulation [8,9,10,11]. The circulating hemocytes of the larva are classified into three categories: the phagocytic plasmatocytes, the melanizing crystal cells, and the encapsulating lamellocytes [9], which are distinguished by the expression of cell-type specific antigens and transgenic markers [12,13,14,15,16,17,18]. Lamellocytes are not present in naive larvae, but they vigorously differentiate in response to various stress stimuli, including infestation by the parasitoid wasp *Leptopilina boulardi*, and eliminate the invader by forming a multilayered melanizing capsule around the parasite egg [19,20,21]. 

The lamellocyte pool that arises upon immune induction originates from all three hematopoietic compartments [9,22]. Lamellocytes differentiate from two separate cell types: plasmatocytes and dedicated precursors [22,23,24,25,26,27]. Plasmatocytes located in the sessile tissue and in the circulation are capable of transdifferentiating into lamellocytes as a result of genetic reprogramming or immune induction [22,24,26,28,29]. So far, dedicated precursor hemocytes, which are predestined to become lamellocytes, were found exclusively in the lymph gland [25]. Precursor hemocytes are located in the medullary zone of the lymph gland and are under the control of the posterior signaling center (PSC), a group of cells distinguished by the expression of the transcription factor Collier [30]. Cells of the PSC emanate cytonemes into the medullary zone. Signaling from the PSC is essential to maintain the prohemocyte state of medullary zone cells, therefore the PSC is regarded as a genuine hemocyte niche [31,32]. Concomitantly, alteration of PSC structure or activity was described to trigger precocious differentiation of the medullary zone population into various effector cell types [31,32,33,34,35]. However, recent results demonstrated that the PSC is not directly required for the maintenance of undifferentiated cells in the medullary zone (MZ), rather it controls their proper differentiation upon an immune challenge through relaying both activating and suppressive signals toward the progenitor cells [36,37].

In the lymph gland, prohemocyte state and effector hemocyte differentiation are controlled by a complex signaling network. Major components of this network that contribute to hemocyte fate choice are the Hedgehog (Hh), the Decapentaplegic (Dpp), and the JAK/STAT signaling pathways [11,32,38,39,40].

Hh and Dpp signaling originates from the PSC and is required to maintain the undifferentiated state of the MZ non-autonomously [32,41]. Active JAK/STAT signaling is required in the MZ cells to block their premature differentiation. Concomitantly, JAK/STAT activity is downregulated in these cells upon immune challenge to enable the differentiation of effector hemocytes [42,43,44]. Furthermore, JAK/STAT has been also implicated to act in the CZ cells to control plasmatocyte differentiation [45]. In unchallenged larvae, the lymph gland remains separated from the circulation until pupation, i.e., no hemocyte can enter or leave the organ [22,46]. However, following immune induction, effector hemocytes, including lamellocytes, differentiate from the progenitor cells and enter the circulation [22,23].

During lamellocyte differentiation, hemocytes gradually change their morphology and gene expression patterns [24,28,29]. A portion of the genes that becomes activated or silenced during the differentiation process contributes to the effector function of the cells, e.g., genes encoding phagocytosis receptors such as NimC1 and Eater [22,27,47] are downregulated. Other markers that are expressed in different hemocyte types represent regulator genes, such as transcription and epigenetic factors that directly determine the fate of the given cell type [9].

In this study, we investigated the role of Headcase (Hdc), the *Drosophila* homolog of HECA (Hdc homolog, cell cycle regulator), in the regulation of hematopoiesis. HECA interacts with cyclins and acts as a suppressor of several types of tumors in humans [48,49,50,51]. Our previous results [28], in accord with earlier literature data [52], showed that *hdc* is expressed in the cells of the lymph gland. However, the effector hemocytes that differentiate and exit the lymph gland upon immune induction showed no *hdc-LacZ* expression [28], suggesting that Hdc activity may be related to the differentiation state of hemocytes. Hdc was originally described as a factor that blocks premature differentiation of imaginal tissues [52], and it was later shown to be active as a maintenance factor in the stem cell niche of the testis [53]. Hdc was also identified as a marker of intestinal stem cells and enteroblasts [54]. Nevertheless, the molecular function of Hdc is still unknown, and no characteristic domains were identified in the protein. Our results show that *hdc* expression in the lymph gland is developmentally regulated, and Hdc plays an indispensable role in blocking premature lamellocyte differentiation. Hdc acts in the PSC, upstream of the Hedgehog and Decapentaplegic regulatory pathways, which normally maintain the prohemocyte state of medullary zone cells.

## 2. Materials and Methods

### 2.1. Drosophila Stocks

The following *Drosophila* lines were used in the study: *w^1118^* (BSC#9505), *w; P{GawB}5015* (BSC#2721), *w; hdc^B5^* (a gift from Christos Samakovlis), *w; hdc^19^-Gal4/TM3, Kr>GFP* (this study), *w; hdc^43^/TM6, Tb (BSC#64063)*, *w; hdc^Δ84^/TM3, Kr>GFP* (this study), *w; HmlΔ-Gal4, UAS-GFP* [55], *w; UAS-hdcRNAi (VDRC#v45069)*, *w; HmlΔ-Gal4, UAS-GFP, UAS-hdcRNAi*, *w; Dot-Gal4* (BSC#67608), *y, w, UAS-FLP; Dot-Gal4, AFG, UAS-GFP* [22], *w; Dot-Gal4,UAS-hdcRNAi*, *w; Pcol85-Gal4/CyO, GFP* [31], *w; Pcol85-Gal4, UAS-hdcRNAi/CyO, GFP*, *y, w; UAS-2xEGFP (BSC#6658)*, *w; Sp/CyO; UAS-hh(M4)/TM6b* (a gift from Tamás Matusek), *w; UAS-dpp (BSC#1486)*, *w; UAS-hepRNAi* (VDRC#v47507)*, w; UAS-hopRNAi* (VDRC#v102830)*, w; UAS-hdc.S* (a gift from Christos Samakovlis), *w; UAS-hdc.S* (2nd chromosomal insertion, generated by the remobilization of the P element in *BSC#6658*), *w; hdc^19^-Gal4, UAS-hdc.S/TM3* (this study).

The flies were kept on a standard cornmeal-yeast diet at 25 °C. All crosses were performed at 25 °C.

### 2.2. Antibodies

Lamellocytes were detected with a mixture of L1a, L1b, and L1c (L1) mouse monoclonal antibodies [15]. Plasmatocytes were stained with the mixture of P1a and P1b (P1) antibodies [14]. PSC cells were stained with anti-Collier antibody [30], a kind gift from Michele Crozatier. The bound primary antibodies were visualized with CF 568 conjugated goat anti-mouse immunoglobulin (Biotium, Cat: 20100).

### 2.3. P Element Conversion

The exchange of the enhancer trapping P element was carried out according to Sepp and Auld [56]. Briefly, *w; P{GawB}/SM6b; P{LacZ}hdc^B5^/TM3*Δ*2-3* jumpstarter virgins were crossed to *w^1118^* in order to increase the likelihood of the successful conversion events. Single *w; SM6b/+; P{GawB}hdc/+* male progeny were crossed to *w^1118^* virgins to map the insertions to chromosomes based on the segregation of markers. Insertions segregating irrespectively of *SM6b* and sex chromosomes were regarded as third chromosomal. Candidate males were crossed individually to *y, w; UAS-2xEGFP* virgins to verify the Gal4 activity and expression pattern.

### 2.4. X-GAL Staining

Lymph glands and imaginal discs were dissected from wandering larvae in PBT on ice, then were fixed and stained as described by Jankovics et al. [57].

### 2.5. PCR Mapping of the P Element Insertions

The localization of the *P{LacZ}hdc^B5^* element was determined by screening with a set of forward primers covering the *hdc* genomic region and a reverse primer specific for the P element. Genomic DNA was isolated from female adult flies using the GenEluteTM Mammalian Genomic DNA Miniprep Kit (Sigma). PCR reactions of 100 ng genomic DNA template and the primer set *Insertion forward* (5’-CGAGCCGCAACGAAAGTG-3’) and *Insertion reverse* (5’-CCACCTTATGTTATTTCATCATG-3’) were found to amplify a 701 bp DNA fragment.

The fragment was isolated and sequenced using a BigDye Terminator v3.1 Cycle Sequencing Kit (Invitrogen) and a 3500-Genetic Analyzer (Applied Biosystems), with the primer *FarHdc fw* (5’-TGAAGAAGTGCGGAAAATCGG-3’). The sequencing revealed that the *P{LacZ}hdc^B5^* insertion is localized 1017 bp upstream of the *hdc* start codon.

A similar strategy was used to determine the position of the *P{GawB}hdc* insertions in three independent convertants (*hdc^19^-Gal4*, *hdc^31^-Gal4*, *hdc^55^-Gal4*). PCR reactions using the *hdc rev* (5’-TCCCACCACTCGAAGCACTC-3’) and *PGawB end* (5’-GCTATGACCATGATTACGCCAAG-3’) primer pair amplified a 1748 bp DNA fragment. The fragment was isolated and sequenced with the *PGawB end* primer. In each tested line, the *P{GawB}* insertion was localized 1025 bp upstream of the *hdc* start codon.

### 2.6. Generation of hdc Alleles and Breakpoint Mapping

Jumpstarter males (*w; hdc^19^-Gal4/TM3, Δ2-3, ry^+^*) were crossed to *TM6/TM3* virgins. Candidates were selected for the loss of the *miniwhite* marker gene and for lethality in combination with the *hdc^31^-Gal4* insertion, an independent hypomorphic mutation isolated in our previous P element conversion screen.

We carried out a PCR screening on the candidate lines with the *Excision forward* (5’-ACCAATCTCGGTTAGAAACCCACT-3’) and the *Excision reverse* (5’-TCCCACCACTCGAAGCACTC-3’) primers, which amplify an 823 bp long fragment overlapping the translation start codon of *hdc*, to isolate amorphic alleles, in which the protein coding region of the *hdc* gene is affected by the deficiency. The candidate that did not yield the expected PCR fragment was further analyzed with various primer sets. The breakpoints of the deficiency were identified with the *Upstream forward* (5’-ACCAAATTCTGGCCTACAGTGG-3’) and *Upstream reverse* (5’-CCACCTTATGTTATTTCATCATG-3’) primers for the upstream breakpoint, and *Downstream forward* (5’-TGGCATCATTGAAACAGCAAGG-3’) and *Downstream reverse* (5’-GGATATCTCGCCACTGGACTG-3’) primers for the downstream breakpoint.

### 2.7. Immunostaining of Circulating Hemocytes, Imaging, and Counting

Larvae were dissected in 30 µl Schneider’s medium (Lonza) supplemented with 5% FBS (Gibco) and n-Phenylthiourea (PTU, Sigma-Aldrich) on a multispot microscope slide (Hendley-Essex SM011). The isolated hemocytes were allowed to adhere at room temperature for one hour. The samples were fixed with acetone for 6 min or with 2% paraformaldehyde for 10 min, washed three times with PBS and blocked for 20 min in PBS containing 0.1% BSA (Roche Diagnostics GmbH), incubated with primary antibodies for 45 min at room temperature, washed three times in PBS, and stained with CF 568-conjugated goat anti-mouse antibody (Biotium) for 45 min. Nuclei were visualized with DAPI (Sigma-Aldrich). The samples were mounted with 1:1 PBS-glycerol or Fluoromount-G (SouthernBiotech). For quantifications, hemocytes were identified with DAPI staining, lamellocyte and plasmatocyte ratios were determined with ImageJ, based on lamellocyte specific L1 staining and plasmatocyte specific P1 staining, respectively. Statistical analysis and graph assembly were performed using GraphPad Prism 6.

### 2.8. Preparation and Immunostaining of the Lymph Gland

Lymph glands of the larvae were dissected, fixed, and blocked as described by Márkus et al. [28]. The samples were incubated with respective monoclonal antibodies overnight at 4 °C, washed three times in PBS, and stained with CF 568-conjugated goat anti-mouse antibody at room temperature for 45 min. Nuclei were stained with DAPI (Sigma-Aldrich). Samples were mounted with Fluoromount-G (SouthernBiotech).

## 3. Results

### 3.1. hdc is Expressed in the Lymph Gland, but Not in the Other Hemocyte Compartments

**Figure 1:**
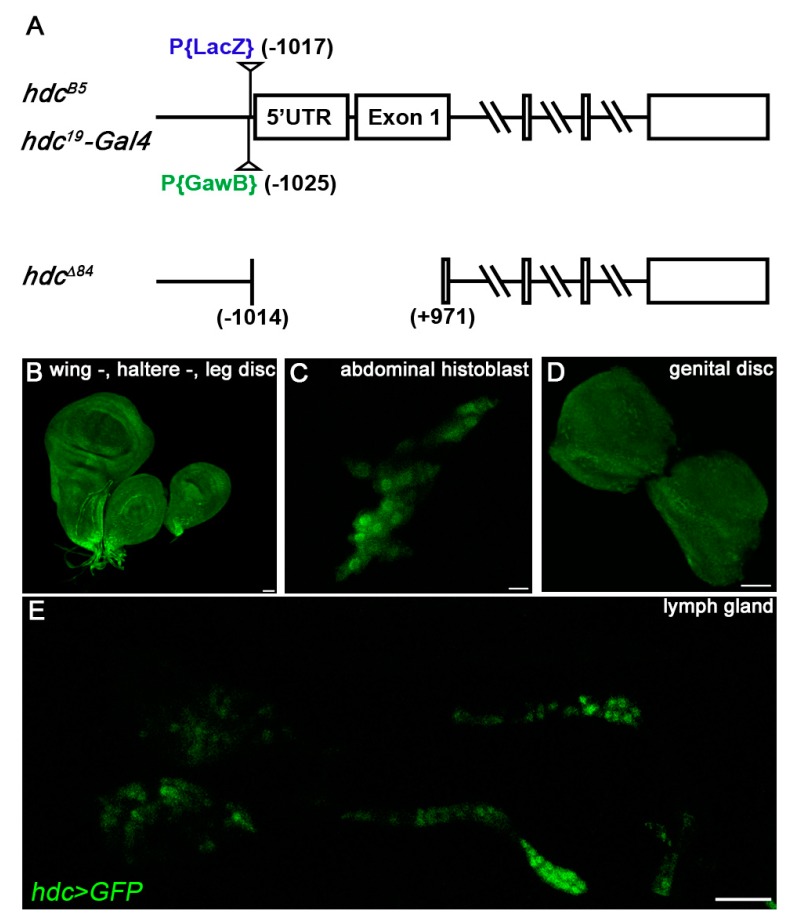
Genomic locations of *hdc* alleles and the expression pattern of *hdc*>*GFP*. (**A**) Molecular map of the *hdc* gene region. The positions of the original *hdc^B5^* insertion, the *hdc^19^-Gal4* insertion, and the breakpoints of the *hdc^Δ84^* deletion are indicated; (**B**–**E**) *hdc^19^-Gal4>GFP* (green) was expressed in the imaginal discs (**B**), the abdominal histoblasts (**C**), the genital disc (**D**), and in the lymph gland (**E**) of the larva. Images were taken with Leica TCS SP5 Confocal Microscope, original magnification ×20 (**B**,**E**) and ×40 (**C**,**D**), and processed with LAS AF Lite (Leica Microsystems CMS GmbH), ImageJ and Adobe Photoshop CS2 software. Scale bars: 25 μm.

To enable the thorough analysis of *hdc* expression and functional studies in vivo, we performed a P element conversion screen, in which the *LacZ* containing P element (*P{LacZ}hdc^B5^*) in the *hdc^B5^* allele was exchanged with a *Gal4* containing *P{GawB}* enhancer trap element. This was achieved by the simultaneous mobilization of the two P elements [56]. Of the several *hdc-Gal4* lines, we selected *hdc^19^-Gal4* for further analysis. As detected by X-Gal staining, *hdc^19^-Gal4* did not show LacZ expression in any tissues or organs of the larva, confirming that the *P{LacZ}hdc^B5^* element was completely removed from the genome. Molecular analysis revealed that the *P{GawB}* insertion in the *hdc^19^-Gal4* allele was located 8 bp upstream of the original *P{LacZ}* insertion (1025 bp upstream of the translation start codon of the gene) (Figure 1A).

*hdc^19^-Gal4>GFP* larvae (*hdc^19^-Gal4/UAS-2xEGFP*) expressed GFP in the imaginal discs, the abdominal histoblasts, the genital disc, and the lymph gland (Figure 1B–E), similarly to the LacZ expression of the *hdc^B5^* larvae. No GFP expression was detected in adult flies, which underlines that Hdc is required in the larval stages for the formation of adult tissues [52]. These results indicate that the *P{GawB}hdc^19^-Gal4* element traps the same enhancers as the *P{LacZ}hdc^B5^* insertion. GFP expression was not detected in the circulating and sessile hemocytes of the *hdc^19^-Gal4>GFP* larvae, showing that *hdc* is not expressed in hemocytes outside of the lymph gland.

### 3.2. hdc Enhancer Trap Insertions are Hypomorphic hdc Alleles

Although *hdc^B5^* was homozygous viable, *hdc^19^-Gal4* showed pupal lethality both in homozygous condition, and in combination with two other *P{GawB}hdc* insertions (*hdc^31^-Gal4, hdc^55^-Gal4*), which were isolated in the same conversion screen and were located in the same molecular position. Similarly, *hdc^19^-Gal4* was lethal in combination with *hdc^43^*, a previously described null mutant allele [52], but it was viable in combination with *hdc^B5^*. These findings suggest that *hdc^19^-Gal4* is a stronger hypomorphic allele than *hdc^B5^*. The most obvious explanation for this phenomenon may be the possibly stronger enhancer trapping activity of the *P{GawB}hdc^19^-Gal4* than that of the original *P{LacZ}hdc^B5^* element.

Homozygous lethality of the *hdc^19^-Gal4* insertion was rescued by the expression of *hdc* from a *UAS-hdc* transgene (*hdc^19^-Gal4/hdc^19^-Gal4, UAS-hdc.S*), indicating that lethality is not caused by a second-site mutation. Additionally, it confirmed that Gal4 expression in the *hdc^19^-Gal4* recapitulates the endogenous *hdc* expression pattern (Figure 1B–E). RNA-interference mediated silencing of *hdc* with the *hdc^19^-Gal4* (*hdcRNAi/+; hdc^19^-Gal4/+*) resulted in 100% pupal lethality, which is typical to *hdc* loss-of-function [52].

### 3.3. hdc Loss-Of-Function Mutant Larvae Produce Lamellocytes without Immune Induction

**Figure 2:**
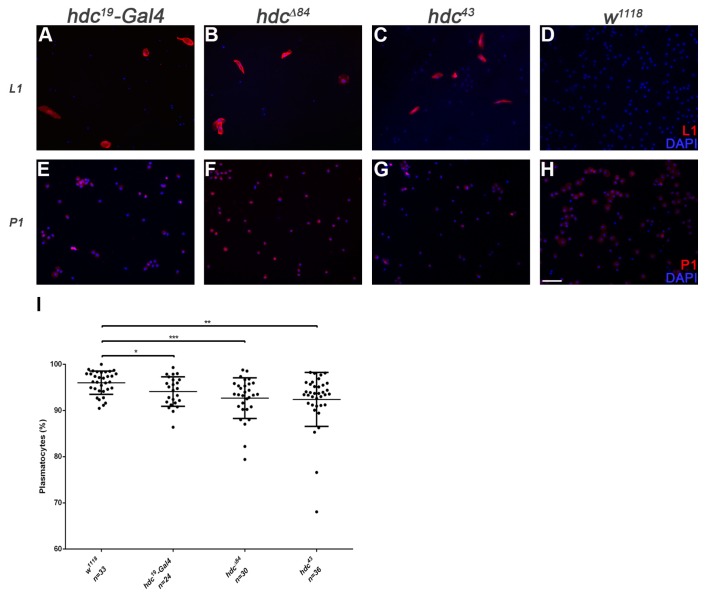
Circulating hemocytes of the *hdc* mutant larvae. (**A**–**D**) Lamellocytes were present in the circulation of *hdc^19^-Gal4* (2.61% (n = 42)) (**A**), *hdc^∆84^* (2.15% (n = 24)) (**B**) and *hdc^43^* (6.07% (n = 52)) (**C**) homozygous mutant larvae. Lamellocytes were rarely observed in control *w^1118^* larvae (0.07% (n = 24)) (**D**). Lamellocytes were stained with L1 antibody (red); (**E**–**H**) Reduced plasmatocyte ratio was observed in *hdc^19^-Gal4* (**E**), *hdc^Δ84^* (**F**), and *hdc^43^* (**G**) homozygous mutant larvae compared to control *w^1118^* (**H**). Plasmatocytes were stained with P1 antibody (red). Nuclei were stained with DAPI (blue). Hemocytes were visualized on a Zeiss Axioskope 2MOT epifluorescent microscope, original magnification ×10 and processed with ImageJ and Adobe Photoshop CS2 softwares. Scale bar: 20 μm. (**I**) Quantification of plasmatocytes in the *hdc* mutant larvae. Error bars represent the standard deviation of the mean. ***: *p* < 0.001; **: *p* < 0.01; *: *p* < 0.05. Significance was determined by unpaired Student’s *t*-test.

We analyzed the hematopoietic phenotype of *hdc* by staining the circulating hemocytes of third instar homozygous *hdc^19^-Gal4* larvae for the P1 marker of plasmatocytes [14] and the L1 marker for lamellocytes [15]. Immunostainings revealed the precocious differentiation of lamellocytes in homozygous *hdc^19^-Gal4* larvae with 19% penetrance (Figure 2A).

To elucidate the effects of complete loss of *hdc*, we generated loss-of-function *hdc* mutants with the remobilization of the P element in the *hdc^19^-Gal4* line. Of the 13 isolated lethal alleles, molecular mapping of the *hdc* locus uncovered one deletion, *hdc^Δ84^*, in which the start codon was deleted together with a part of the first exon, spanning 1014 bp upstream and 971 bp downstream of the ATG site (Figure 1A). Considering the original position of the *P{GawB}* element, we conclude that the P element underwent a local hop of 11 bp before generating the excision. The newly generated *hdc^Δ84^* allele was pupal lethal both in homozygous condition and in interallelic combination with *hdc^43^*, which was described as a null allele [52]. Based on these data, *hdc^Δ84^* can be also regarded as a null allele of the gene.

**Figure 3:**
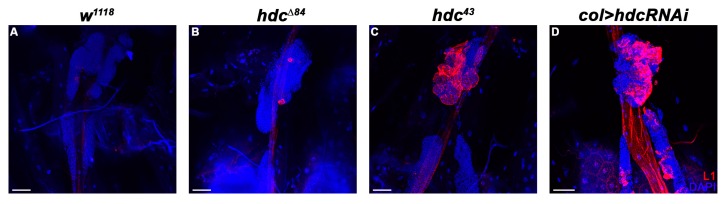
Lamellocyte differentiation in the lymph gland of *hdc* mutant and *hdc* silenced larvae. (**A**) There are no lamellocytes present in the lymph gland of *w^1118^* control larvae; (**B**–**C**) Lamellocytes are detected with staining for the L1 marker (red) in the lymph gland of *hdc^Δ84^* (**B**) and *hdc^43^* (**C**) larvae; (**D**) Silencing of *hdc* with the *col-Gal4* driver (*Pcol85-Gal4, UAS-hdcRNAi/+*) also resulted in the differentiation of lamellocytes (red) in the lymph gland. Nuclei were stained with DAPI (blue). Confocal images were generated with a Leica TCS SP5 Confocal Microscope, original magnification ×20, and processed with LAS AF Lite (Leica Microsystems CMS GmbH), ImageJ and Adobe Photoshop CS2 softwares. Scale bars: 50 μm.

Immunostaining of circulating larval hemocytes for the L1 marker showed that both *hdc^Δ84^* and *hdc^43^* homozygous larvae contained lamellocytes with 83% and 100% penetrance, respectively (Figure 2B–D). Lamellocyte differentiation was also observed in the lymph gland (Figure 3). The ratio of plasmatocytes was significantly reduced in larvae carrying homozygous *hdc* alleles (Figure 2E–I), which may be due to the appearance of lamellocytes in the circulation.

### 3.4. Hdc Exerts its Effect in the Lymph Gland

**Figure 4:**
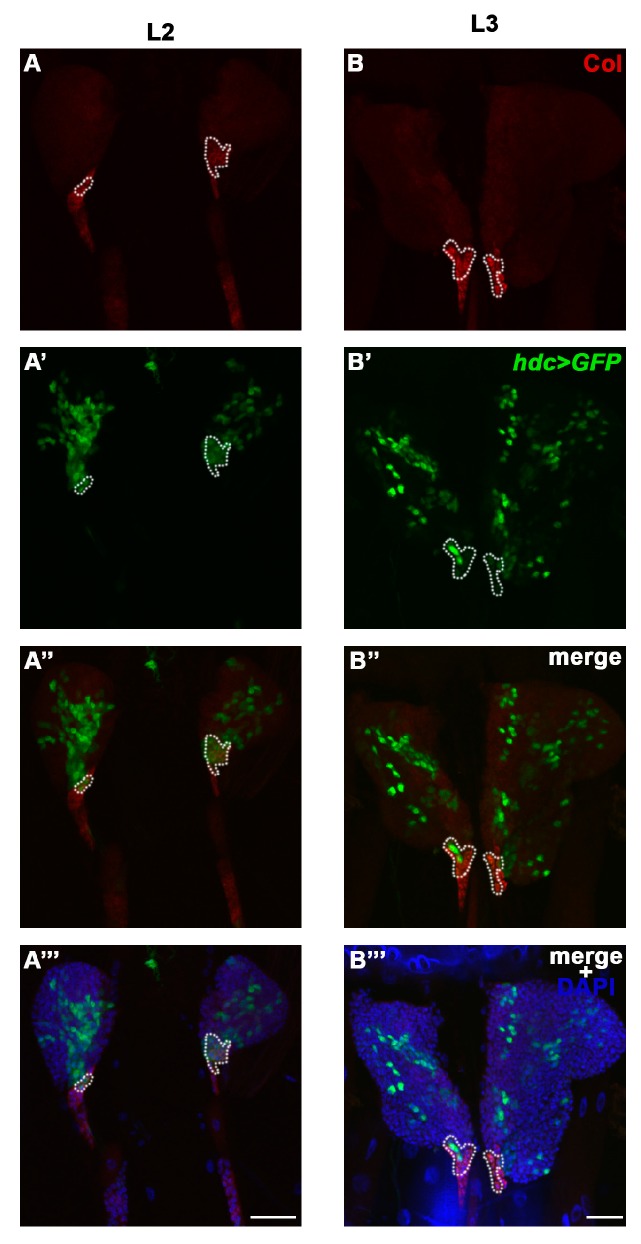
Overlap of *hdc>GFP* and Collier expression in the posterior signaling center (PSC) of the lymph gland. (**A**–**B**) PSC was labeled with anti-Collier staining (red) of the lymph gland of L2 (**A**) and L3 (**B**) larvae; (**A’**–**B’**) The expression of *hdc* was monitored by the expression of the GFP marker (green) in second- (**A’**) and third instar (**B’**) *hdc19-Gal4/UAS-2xEGFP* larvae; (**A’’**–**B’’**) *hdc>GFP* (green) and Collier (red) expressions overlap in the PSC of the lymph gland; (**A’’’**–**B’’’**) DAPI staining (blue) of lymph gland cells. Images were taken with a Leica TCS SP5 Confocal Microscope, original magnification ×20, and processed with LAS AF Lite (Leica Microsystems CMS GmbH), ImageJ and Adobe Photoshop CS2 softwares. Scale bars: 25 μm.

**Figure A1:**
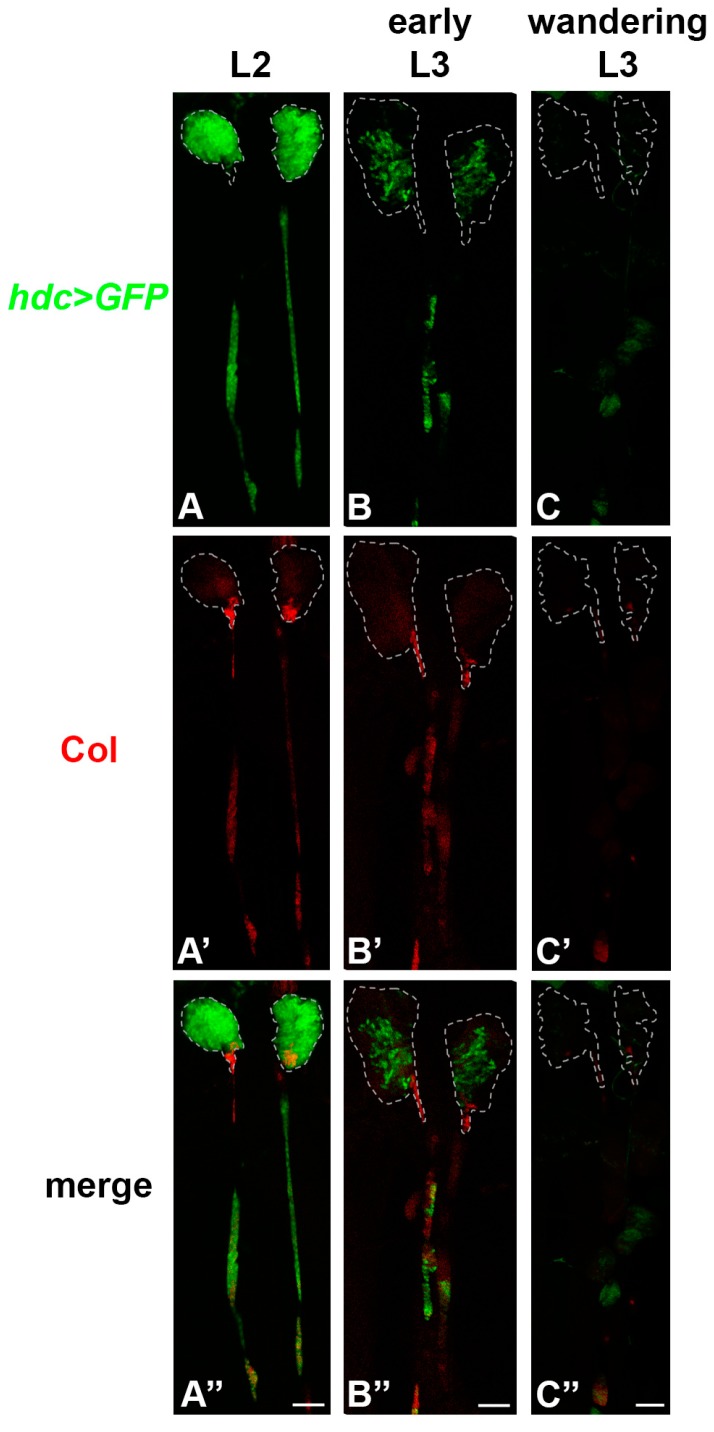
*hdc*>*GFP* expression in the lymph gland. (**A**–**C’’**) Confocal images of lymph glands of second instar (**A,A’,A’’**), early third instar (**B,B’,B’’**) and wandering (**C,C’,C’’**) *hdc^19^-Gal4/UAS-2xEGFP* larvae; (**A**) *hdc^19^-Gal4>GFP* (green) was expressed throughout the primary lymph gland lobes in 2nd instar larvae; (**A’**) Anti-Collier staining (red) marked the PSC of the lymph gland, and most of the secondary lobe cells; (**A’’**) The GFP expression (green) partially overlapped with anti-Collier staining (red); (**B**) In early 3rd instar larvae, the number of *hdc^19^-Gal4>GFP* expressing cells (green) were greatly reduced in the primary lobes of the lymph gland; (**B’,B’’**) The overlap of *hdc^19^-Gal4>GFP* (green) and anti-Collier staining (red) was observable only in the secondary lymph gland lobes, but not in the PSC; (**C**) In wandering 3rd instar larvae, the number of *hdc^19^-Gal4>GFP* expressing lymph gland cells was further reduced; (**C’,C’’**) Virtually no overlap of *hdc^19^-Gal4>GFP* (green) and Collier expressing (red) cells was detected. Dashed lines indicate the primary lobes of the lymph glands. Images were taken with Leica TCS SP5 Confocal Microscope, original magnification ×40 (**A**–**A’’**,**B**–**B’’**) and ×20 (**C**–**C’’**), and processed with LAS AF Lite (Leica Microsystems CMS GmbH), ImageJ and Adobe Photoshop CS2 softwares. Scale bars: 25 μm.

To locate the focus of the *hdc* mutation in relation to its hematopoietic phenotype, first we analyzed the *hdc^19^-Gal4>GFP* expression in the lymph gland at different larval stages. In L2 larvae, *hdc^19^-Gal4>GFP* was broadly expressed in the primary lobes of the lymph gland. The expression pattern partially overlapped with the anti-Collier staining in the PSC and in the secondary lymph gland lobes (Figure 4A–A’’’ and Figure A1A–A’’). In early L3 larvae, the expression pattern was greatly altered as compared to the L2 stage, the *hdc^19^-Gal4>GFP* being expressed in less hemocytes of the primary lobes, and only in a few of the cells in the PSC (Figure 4B–B’’’ and Figure A1B–B’’). In wandering L3 larvae, the number of GFP expressing hemocytes was further reduced in the primary lobes, and no GFP expression was detected in the PSC. However, *hdc^19^-Gal4>GFP* was still detectable in a subset of the cells in the secondary lymph gland lobes (Figure A1C–C’’).

**Figure 5:**
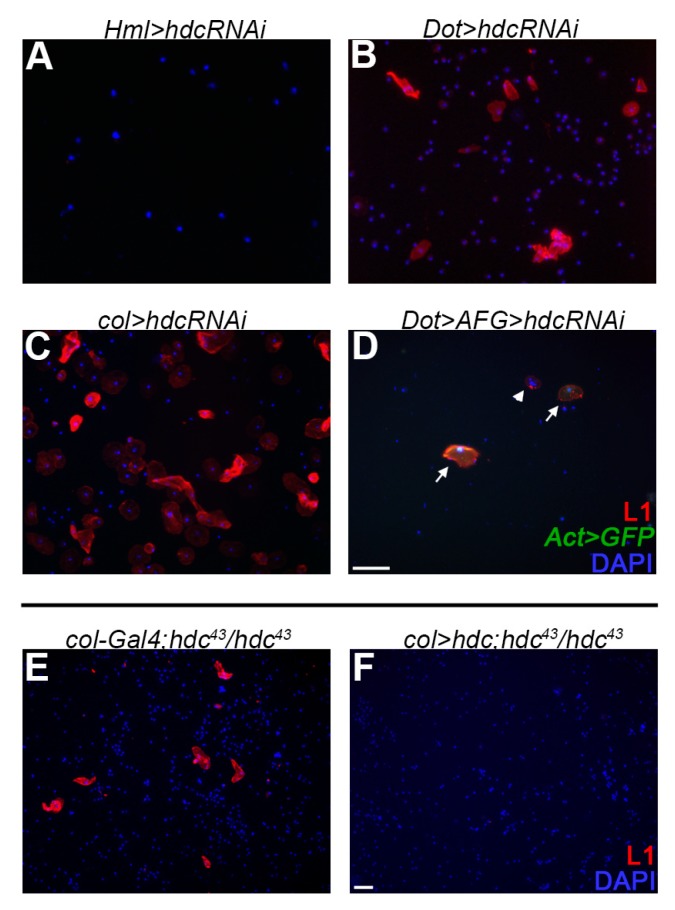
The effect of *hdc* silencing and the rescue of the hematopoietic phenotype. (**A**) Silencing *hdc* with the plasmatocyte specific *Hml-Gal4* (*HmlΔ-Gal4, UAS-GFP, UAS-hdcRNAi*) did not result in the differentiation of lamellocytes; (**B**) Silencing *hdc* with the lymph gland specific *Dot-Gal4* driver (*Dot-Gal4/UAS-hdcRNAi*) resulted in the differentiation of lamellocytes (red); (**C**) Silencing *hdc* in the PSC of the lymph gland with the *col-Gal4* driver (*Pcol85-Gal4/UAS-hdcRNAi*) led to lamellocyte differentiation (red); (**D**) Silencing of *hdc* in the entire *Dot*-lineage (*y, w, UAS-Flp; Dot-Gal4, AFG/UAS-hdcRNAi*) resulted in the appearance of lamellocytes in the circulation (red). These lamellocytes fell into two classes. The lamellocytes, which derive from the *Dot*-lineage, expressed both GFP (green) and the lamellocyte specific L1 antigene (red). The lineage-independent lamellocytes expressed only the L1 marker (red). Arrowhead indicates a GFP^-^ lamellocyte, while arrows point to double positive lamellocytes; (**E**–**F**) Lamellocytes (red) were always present in the circulation of *hdc^43^* homozygous larvae (*Pcol85-Gal4/+; hdc^43^/hdc^43^*) (2.85% (n = 12)) (**E**), while their proportion was reduced when *hdc* was overexpressed with the *col-Gal4* driver (*Pcol85-Gal4/UAS-hdc.S; hdc^43^/hdc^43^*) (0.25% (n = 12)) (**F**). Nuclei were stained with DAPI (blue). Hemocytes were visualized on a Zeiss Axioskope 2MOT epifluorescent microscope, original magnification ×20 (**A**–**D**) and ×10 (**E**,**F**) and processed with ImageJ and Adobe Photoshop CS2 softwares. Scale bars: 20 μm.

To determine the subgroup of hemocytes in which Hdc is required, we silenced *hdc* with various drivers. Silencing *hdc* with *Hemolectin-Gal4* (*HmlΔ-Gal4*) [55], a general plasmatocyte specific driver, which is active in all three hemocyte compartments, but inactive in the PSC, did not result in lamellocyte differentiation. However, silencing *hdc* with the lymph gland specific *Dorothy-Gal4* (*Dot-Gal4*) [58] or *col-Gal4* (*Pcol85-Gal4*) [31] led to the appearance of lamellocytes in the circulation. As the overlapping domain of expression of these two drivers is the PSC, we concluded that lamellocyte differentiation is triggered upon loss of *hdc* function in the hematopoietic niche of the lymph gland (Figure 5A–C). Importantly, overexpression of *hdc* with the *col-Gal4* driver rescued the hematopoietic phenotype of *hdc^43^* homozygous larvae (Figure 5E,F).

As these findings suggested that Hdc has a non-cell-autonomous function in the PSC to regulate the fate of the medullary zone hemocytes, we knocked down *hdc* with RNA interference in the *Dot* hemocyte lineage, which is composed of cells from all the three functional zones of the lymph gland [22,59], and scored circulating immune cells for cell-type specific markers. We found that 68% of the lamellocytes (n = 66) appearing in third instar larvae were derived from cells that did not belong to the *Dot*-lineage (Figure 5D), showing that lamellocyte differentiation mostly occurred in a non-lineage-autonomous fashion. These results, together with the appearance of lamellocytes when *hdc* is knocked down specifically in the PSC, suggest that Hdc is required in the signaling center for its capacity to negatively regulate lamellocyte differentiation in the lymph gland.

### 3.5. Hdc Interacts with the Hedgehog and the Decapentaplegic Pathways

**Figure 6:**
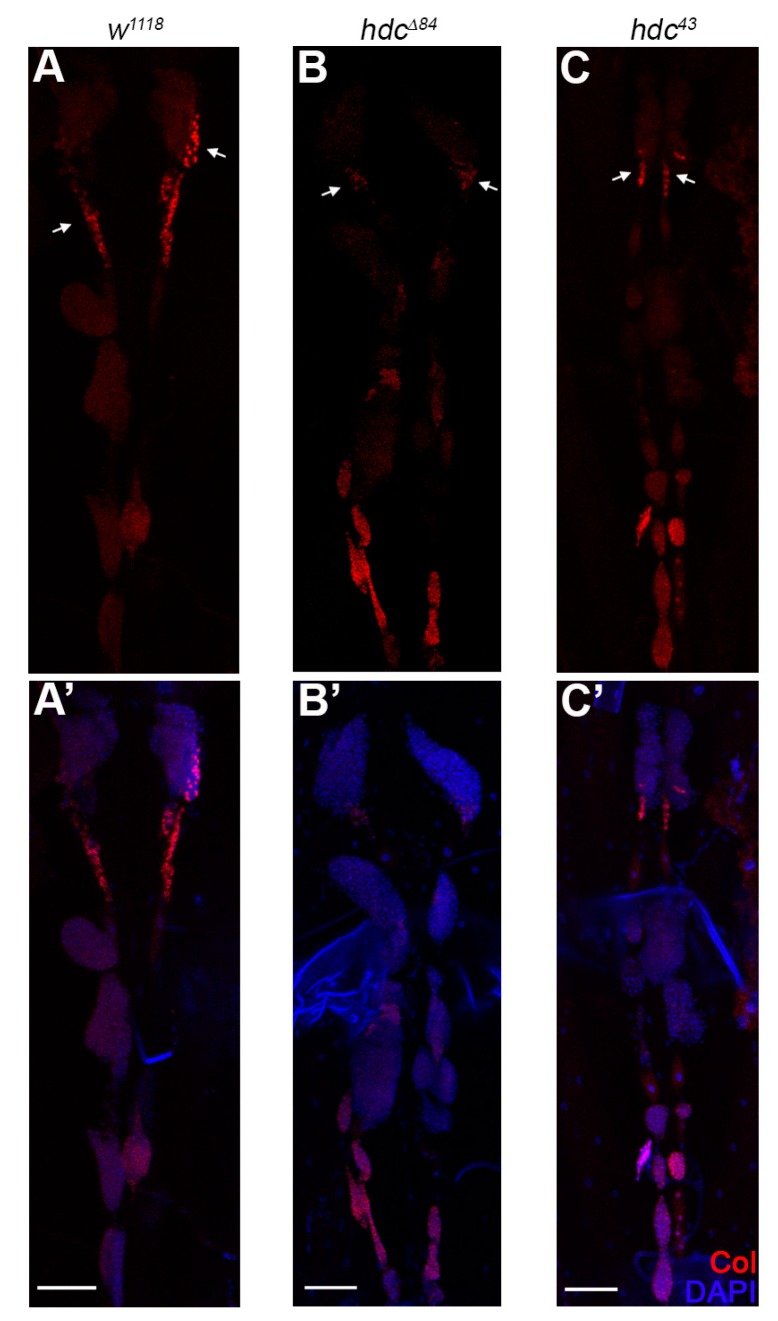
Integrity of the lymph gland and the PSC in *hdc* mutants. (**A**,**A’**) Anti-Collier staining (red) of the lymph gland of the *w^1118^* control larva; (**B**–**C’**) Anti-Collier staining (red) of the lymph gland of the *hdc^Δ84^* (**B**,**B’**) and *hdc^43^* (**C**,**C’**) mutant larvae. The size and morphology of the lymph gland and the PSC in the *hdc* mutants did not differ from the control. Nuclei were stained with DAPI (blue) (**A’**,**B’**,**C’**). Arrows point to the PSC of the lymph gland. Images were taken with Leica TCS SP5 Confocal Microscope, original magnification ×20, and processed with LAS AF Lite (Leica Microsystems CMS GmbH), ImageJ and Adobe Photoshop CS2 softwares. Scale bars: 50 μm.

**Figure A2:**
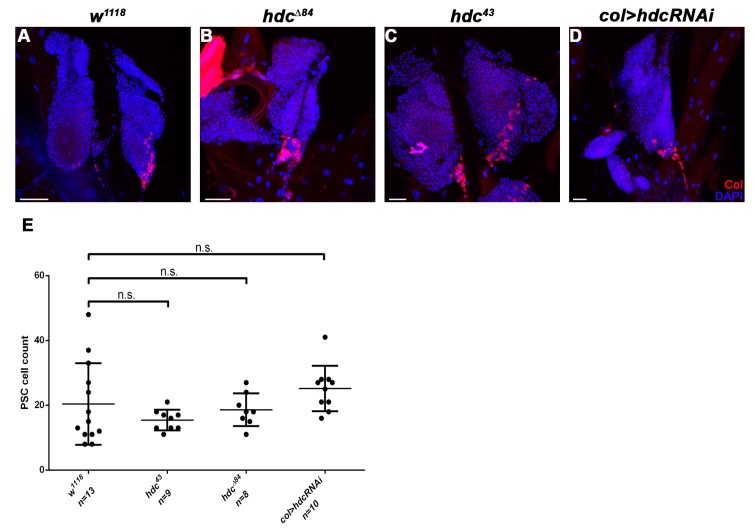
PSC integrity in *hdc*-mutant and *hdc*-silenced larvae. (**A**–**D**) PSC integrity in *w^1118^* control larvae (**A**) is similar to that observed in the *hdc^Δ84^* (**B**) and *hdc^43^* (**C**) mutants. Silencing of *hdc* (**D**) in the PSC with the *col-Gal4* driver (*Pcol85-Gal4, UAS-hdcRNAi/+*) had no effect on the integrity of the PSC. The cells of the PSC (red) were stained with the anti-Collier antibody. Nuclei were stained with DAPI (blue). Images were taken with a Leica TCS SP5 Confocal Microscope, original magnification ×20, and processed with LAS AF Lite (Leica Microsystems CMS GmbH), ImageJ and Adobe Photoshop CS2 software. Scale bars: 25 μm. (**E**) No significant difference was detected in the number of PSC cells in the lymph gland of either of the *hdc*-mutant or the *hdc*-silenced larvae compared to the *w^1118^* control. Error bars represent the standard deviation of the mean. ns: non-significant. Significance was determined by unpaired Student’s *t*-test using GraphPad Prism 6.

As dysfunction of the PSC leads to unprovoked differentiation of lamellocytes in the lymph gland [11,30], we investigated whether the loss of *hdc* affects the integrity or Col expression of the PSC. We found that the size of both the lymph gland and the PSC was unchanged in both the *hdc^43^* and the *hdc^Δ84^* mutant larvae, compared to that of the controls. Similarly, no difference was observed in the expression pattern of Col either in the primary or secondary lymph gland lobes as Col was expressed at a high level in the PSC and in the secondary lobes, and at a low level in the medullary zone of the primary lobes (n = 13 in each genotype). These findings suggest that Hdc is not required in PSC cells to gain or maintain their identity and their Col expression level (Figure 6 and Figure A2).

**Figure 7:**
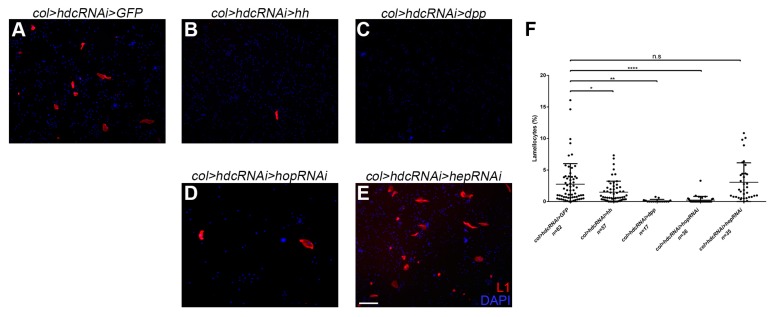
The interaction of Hdc with the signal transduction pathways of the lymph gland. (**A**) Silencing of *hdc* with the *Pcol85-Gal4* driver in the PSC of the lymph gland resulted in differentiation of lamellocytes in naive larvae (*Pcol85-Gal4, UAS-hdcRNAi/+; UAS-2xEGFP/+*); (**B**–**E**) Overexpression of *hh* (**B**) and *dpp* (**C**) with the *col-Gal4* driver significantly reduced the proportion of lamellocytes (red) in the circulation of *hdc*-silenced larvae (*Pcol85-Gal4, UAS-hdcRNAi/UAS-hh(M4)*, *Pcol85-Gal4, UAS-hdcRNAi/UAS-dpp*, respectively). Silencing of *hop* with RNA interference (*Pcol85-Gal4, UAS-hdcRNAi/UAS-hopRNAi*) (**D**) also resulted in the rescue of the *hdc* phenotype, while silencing of *hep* (*Pcol85-Gal4, UAS-hdcRNAi/UAS-hepRNAi*) (**E**) did not affect the phenotype significantly. Nuclei were stained with DAPI (blue). Hemocytes were visualized on a Zeiss Axioskope 2MOT epifluorescent microscope, original magnification ×10 and processed with ImageJ and Adobe Photoshop CS2 softwares. Scale bar: 20 μm. (**F**) Quantification of lamellocyte numbers in the *hdc*-silenced and the rescued larvae. Error bars represent the standard deviation of the mean. **: *p* < 0.01; *: *p* < 0.05; ns: non-significant. Significance was determined by unpaired Student’s *t*-test using GraphPad Prism 6.

In first instar larvae, Dpp signaling maintains niche dependent hematopoietic stem cells (HSCs) in the lymph gland [41], while in later stages, Hh activity is required for the maintenance of the precursor state of medullary zone prohemocytes [32]. To gain an insight into the connection of Hdc to these signaling networks, we silenced *hdc* with the *col-Gal4* driver, and simultaneously, activated Hh and Dpp signaling. The overexpression of both Hh and Dpp significantly reduced the number of circulating lamellocytes in *hdc*-silenced larvae as compared with the controls, indicating that Hdc acts upstream to both signal transduction pathways in the PSC cells (Figure 7A–C,F).

Interestingly, we found that the effect of *hdc* was also partially rescued by the simultaneous silencing of *hopscotch* (*hop*) (Figure 7D,F), a gene coding for the JAK kinase of the JAK/STAT pathway [60]. This pathway was not previously described to be active in the cells of the PSC. Our results, however, suggest that JAK/STAT interacts with Hdc in the regulation of hematopoiesis in the hematopoietic niche of the lymph gland. Silencing of *hemipterous* (*hep*), a serine/threonine protein kinase involved in JNK signaling [61], did not significantly reduce the number of lamellocytes (Figure 7E,F).

## 4. Discussion

In this report, we show that Hdc, the *Drosophila* homolog of the human HECA, is a key component in the regulation of HSC function and hematopoiesis in the fruit fly. Of the three larval hemocyte compartments, *hdc* activity is confined to the lymph gland, suggesting that Hdc acts specifically in this hematopoietic organ. It was also observed that lamellocytes downregulate *hdc* expression when leaving the lymph gland [28]. These findings suggest that Hdc may exert a repressive role during hematopoiesis, which is also corroborated by the observation that *hdc* alleles suppress the disruption of the sessile compartment induced by *Toll^10b^* [62].

**Figure 8:**
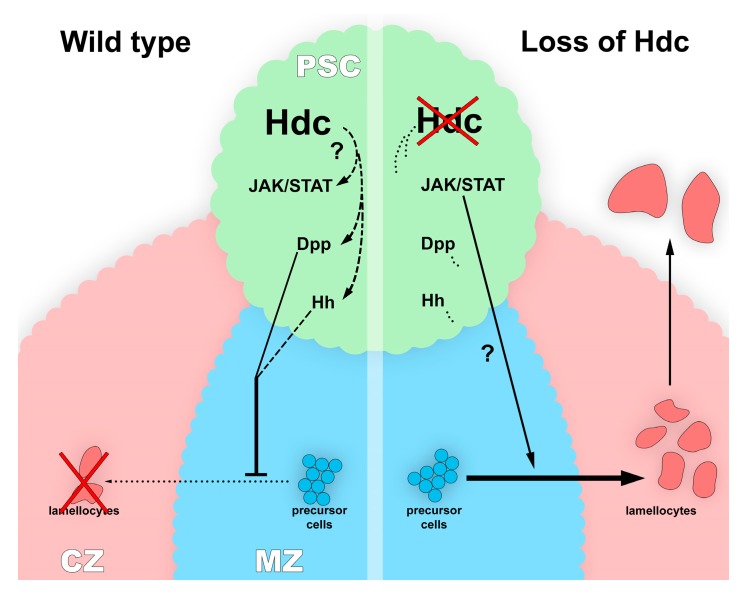
The proposed model of Hdc function in the regulation of signaling pathways in the PSC of the lymph gland. Genetic interactions suggest that Hdc regulates the Hh, the Dpp and the JAK/STAT signaling pathways in the cells of the PSC, thereby maintaining the precursor state of medullary zone (MZ) prohemocytes (left panel). In the lack of Hdc (right panel), the downregulation of the Hh and the Dpp pathways results in the differentiation of lamellocytes in the cortical zone (CZ) without immune induction.

We found that *hdc*>*GFP* is active in the majority of the cells of the lymph gland of second instar larvae, and its expression is gradually switched off at later stages; therefore, we hypothesized that Hdc plays a role in maintaining the non-differentiated state of lymph gland hemocytes. This hypothesis is further underlined by the finding that lamellocytes differentiate in *hdc* loss-of-function mutants, as well as in *hdc*-silenced larvae in a naive state. Although we observed a decreased ratio of plasmatocytes in the circulation in *hdc* mutant larvae, this may correspond to the appearance of lamellocytes, a cell type not observed in control larvae. According to these results, Hdc may play a role in repressing the lamellocyte fate in the lymph gland. It was shown that Hdc is required for the maintenance of the hub cells in the testis niche of larvae [53]. This, together with our data, suggests that Hdc may have a role in maintaining the hemocyte niche function in this organ. While the precise role of the PSC in the developmental control of the lymph gland has recently been challenged [36,37], it is still considered to orchestrate the differentiation of lamellocytes from precursor cells upon immune induction. According to a recent study, the HSC and prohemocyte state of the medullary zone hemocytes is regulated by the consecutive action of the Dpp and the Hh signaling. The Dpp signaling is active only in first instar larva [41], and subsequently its suppressive function is taken over by the Hh pathway [32]. Additionally, it was shown that feeding the larvae with an inhibitor of Smoothened (a key mediator of the Hh signaling pathway) leads to the exit from quiescence in hemocyte progenitors [63]. As the hematopoietic phenotype of *hdc* was suppressed by the overexpression of Hh and Dpp, we speculated that Hdc acts upstream of both these signaling routes. Surprisingly, we also found that Hdc interacts with the JAK/STAT pathway in the PSC (Figure 8). The JAK/STAT pathway was known to regulate lamellocyte differentiation in the larva [40,64,65], however, its effect was not shown previously in the hematopoietic niche of the lymph gland. Our results suggest a complex network of interactions among signaling pathways in the PSC cells, in which JAK/STAT might act antagonistically to Hh and Dpp signaling. Besides the interaction of Hdc with the Hh and Dpp pathways, one possible underlying mechanism of the *hdc* loss-of-function phenotype in the lymph gland may be the generation of reactive oxygen species (ROS), which was previously associated with lamellocyte formation [66]. However, ROS-induced lamellocyte differentiation was also described to cause the disruption of the lymph gland [67], which was not observed in the case of *hdc* loss.

An early study found Hdc to act in a non-cell-autonomous manner to block the formation of branches during trachea development [68]. Likewise, we found that *hdc* silencing in the *Dot*-lineage [22] resulted in the differentiation of lamellocytes from both lineages-traced and lineage-independent precursors. The most rational explanation to this finding is that Hdc activity feeds into communication between the PSC and the precursor cells of the medullary zone.

There is very little information available on how the *hdc* gene is regulated. During tracheal branching, the main regulator of *hdc* expression is *escargot*, a transcription factor acting in stem cells in various *Drosophila* tissue types [68]. It was shown that *buttonhead* and *Sp1* activates *hdc* during the development of ventral imaginal discs [69]. The regulatory region of the *hdc* gene has not yet been characterized, and whether it has a PSC specific enhancer element is unknown. The generated *hdc-Gal4* insertions and their excisions may facilitate the future characterization of these regions.

Hdc is a cytoplasmic protein [52] with a yet unknown molecular function and domain structure. In *Drosophila*, Hdc forms a complex with Unkempt, and functions as a downstream regulator of the InR/mTOR pathway [70]. Our study ascribes a novel function to Hdc in the regulation of hemocyte cell fate via its interaction with the Dpp and Hh signal transduction pathways in the lymph gland. These all suggest that Hdc is an emerging key regulator of HSC maintenance and blood cell differentiation.
